# Metabolic Health, Mitochondrial Fitness, Physical Activity, and Cancer

**DOI:** 10.3390/cancers15030814

**Published:** 2023-01-28

**Authors:** Vicente Javier Clemente-Suárez, Alexandra Martín-Rodríguez, Laura Redondo-Flórez, Pablo Ruisoto, Eduardo Navarro-Jiménez, Domingo Jesús Ramos-Campo, José Francisco Tornero-Aguilera

**Affiliations:** 1 Faculty of Sports Sciences, Universidad Europea de Madrid, Tajo Street, s/n, 28670 Madrid, Spain; 2 Department of Health Sciences, Faculty of Biomedical and Health Sciences, Universidad Europea de Madrid, C/Tajo s/n Villaviciosa de Odón, 28670 Madrid, Spain; 3 Department of Health Sciences, Public University of Navarre, 31006 Navarre, Spain; 4 Facultad de Ciencias de la Salud, Universidad Simón Bolívar, Barranquilla 080001, Colombia; 5 Departamento de Salud y Rendimiento, Universidad Politécnica de Madrid, 28040 Madrid, Spain

**Keywords:** metabolism, mitochondria, oxidative stress, inflammation, physical activity

## Abstract

**Simple Summary:**

Recent research suggests a strong link between the functioning of mitochondria and the development of cancer. Healthy mitochondria play a vital role in maintaining metabolic processes and controlling cell death (apoptosis). However, when inflammation persists for a long period of time, it can harm the mitochondria’s ability to produce energy and manage cells, which in turn leads to tissue damage and hinders regeneration. Engaging in both endurance and resistance exercises, in addition to maintaining an active lifestyle, can help improve the functioning of mitochondria and may decrease the risk of cancer.

**Abstract:**

Cancer continues to be a significant global health issue. Traditional genetic-based approaches to understanding and treating cancer have had limited success. Researchers are increasingly exploring the impact of the environment, specifically inflammation and metabolism, on cancer development. Examining the role of mitochondria in this context is crucial for understanding the connections between metabolic health, physical activity, and cancer. This study aimed to review the literature on this topic through a comprehensive narrative review of various databases including MedLine (PubMed), Cochrane (Wiley), Embase, PsychINFO, and CinAhl. The review highlighted the importance of mitochondrial function in overall health and in regulating key events in cancer development, such as apoptosis. The concept of “mitochondrial fitness” emphasizes the crucial role of mitochondria in cell metabolism, particularly their oxidative functions, and how proper function can prevent replication errors and regulate apoptosis. Engaging in high-energy-demanding movement, such as exercise, is a powerful intervention for improving mitochondrial function and increasing resistance to environmental stressors. These findings support the significance of considering the role of the environment, specifically inflammation and metabolism, in cancer development and treatment. Further research is required to fully understand the mechanisms by which physical activity improves mitochondrial function and potentially reduces the risk of cancer.

## 1. Introduction

**Figure 1:**
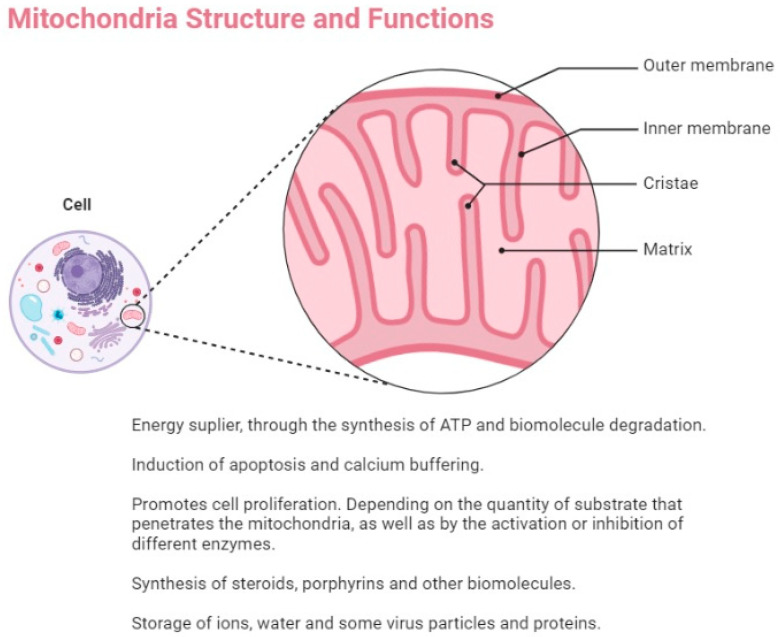
Mitochondria structure and functions.

Mitochondria are essential organelles that perform a wide range of functions within cells, such as producing ATP and metabolic intermediates, as well as assisting in cellular stress responses through processes, such as autophagy and programmed cell death. These organelles are intricately interconnected with other cellular components and are vital for energy metabolism in eukaryotic cells. Specifically, mitochondria generate the majority of the usable energy in cells through the breakdown of carbohydrates and fatty acids through a process called oxidative phosphorylation, which is then converted into ATP (Figure 1).

It was over a hundred years ago when Otto Warburg’s experiments on mitochondrial respiration first highlighted the vital role of mitochondria in eukaryotic cells [1]. In a healthy eukaryotic cell, mitochondria regulate cellular processes, such as metabolic adaptation, calcium homeostasis, and cell proliferation or death. They also participate in reactions, such as the tricarboxylic acid cycle (TCA), fatty acid oxidation (FAO), and oxidative phosphorylation (OXPHOS), which play a role in processes, such as ketogenesis, gluconeogenesis, heme biosynthesis, and Fe/S cluster formation [2]. Additionally, mitochondria contain their DNA, which can be mutated, partially deleted, or damaged due to epigenetic and evolutionary factors. Given the many roles of mitochondria, their potential dysfunction or alteration is closely linked to a wide range of diseases, including cancer. This review, therefore, focuses on metabolic health and the concept of mitochondrial fitness, and how physical exercise can be used as a preventive and adjunctive measure in mitochondrial disease and cancer.

There are five main points that summarize the role of mitochondria in the development of the malignant phenotype through the metabolic reprogramming of cancer cells. First, mutations in mitochondrial DNA can alter subunits of the electron transport chain (ETC) [3]. For example, hepatocellular carcinomas and prostate cancers are associated with mutations in the D-loop region of Complex I, while cancers of neuronal etiology are associated with alterations in succinate dehydrogenase (SDH; Complex II) [4]. Second, reactive oxygen species (ROS) are produced by the mitochondria, with superoxide being a byproduct of oxidative respiration. These ROS include mitochondrial ROS (mtROS), which is produced by the tricarboxylic acid cycle and the electron transport chain [5]. At high concentrations, ROS can be toxic to cellular macromolecules, but at low concentrations, they acts as an intracellular signaling molecule, regulating metabolic pathways. In cancer, high levels of ROS can occur due to excessive metabolic activity and reduced antioxidant capacity [6]. Third, the regulatory role of cell death, apoptosis, and necrosis is important. The B cell lymphoma-2 (Bcl-2) family member proteins interact with mitochondria and bind to the voltage-dependent anion channel (VDAC) to accelerate its opening and the release of cytochrome c, which plays a role in oncogenic or oncosuppressive triggers and cancer progression and therapeutic resistance [7]. Fourth, metabolic reprogramming involves mutations in genes encoding tricarboxylic acid cycle enzymes that promote malignant transformation. Accumulation of tricarboxylic acid cycle enzymes, such as fumarate, succinate, aspartate, and D-2-hydroxyglutarate (2HG) in cells following genetic mutations and/or cancer-associated modifications of protein expression can have pro-carcinogenic effects [2]. Lastly, sustained alterations in cell growth, such as constitutive telomerase expression that maintains telomere length, are characteristic of tumors [8]. Telomerase reverse transcriptase (TERT) shuttles from the nucleus to mitochondria upon oxidative stress, preserving mitochondrial function and decreasing oxidative stress, thus, protecting both mtDNA and nDNA from oxidative damage and preventing apoptosis [2,9].

In summary, the development of cancer is linked to metabolic and mitochondrial alterations, which are closely related to epigenetics, which are largely modifiable factors that can act as triggers for the activation and expression of certain proteins that regulate metabolic pathways related to cancer [10]. In other words, the presence of certain genes does not necessarily cause cancer [11], but rather external conditioning factors, such as epigenetics, determine whether a gene related to cancer is activated or not. For example, over 50% of cancers involve a missing or damaged p53 gene, which are typically acquired mutations. Germline p53 mutations are rare, but patients with them have a higher risk of developing various types of cancer [12]. These disturbances can lead to various health problems, such as chronic fatigue, diabetes, cardiovascular disease, neurodegenerative diseases, migraines, infertility, and cancer. Physical exercise [13] and nutrition [14] are among the epigenetic protectors that can be implemented and modified to improve metabolic and mitochondrial health [15].

**Figure 2:**
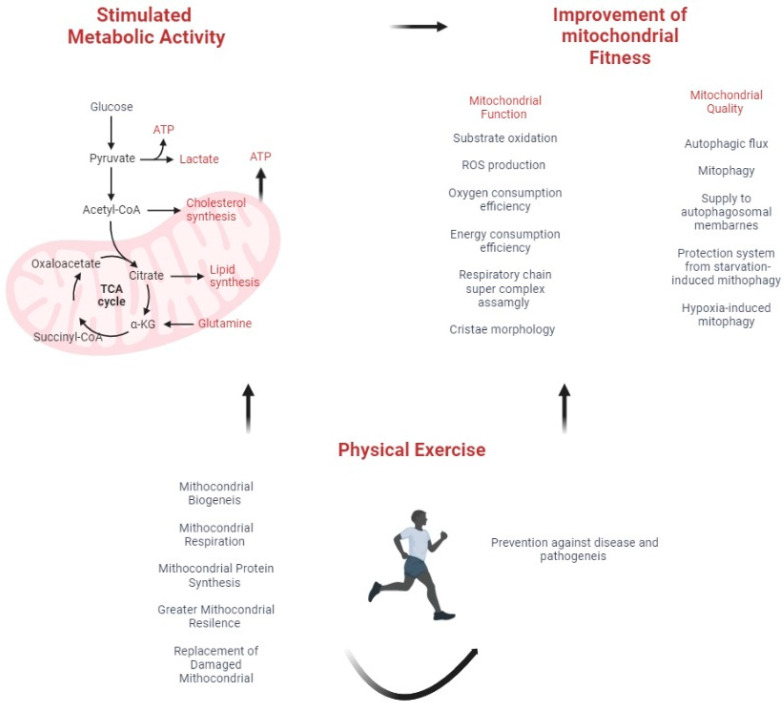
Relationship between metabolic health, mitochondrial fitness, physical activity, and cancer.

In this context, the concept of “mitochondrial fitness” refers to the biological efficiency and adequacy of the mitochondria [16]. According to some authors, this mitochondrial fitness can be divided into function and quality [17], both of which can be improved through physical exercise and nutrition. This narrative review focuses on the role of physical exercise in achieving mitochondrial fitness, which includes mitochondrial biogenesis, mitochondrial respiration, mitochondrial protein synthesis, increased reliance on fatty acid substrates by mitochondria, and better handling of oxidative stress. These processes result in the creation of new mitochondria, increased energy, vitality, and stamina, replacement of damaged mitochondria, prevention of associated diseases, and slower aging [16] (Figure 2).

The aim of this narrative review is to examine the correlation between metabolic health, mitochondrial fitness, physical activity, and cancer. The findings of this study can contribute to a better comprehension of this intricate illness and guide the development of new research methods and clinical interventions.

## 2. Metabolism and Cancer

One of the hallmark characteristics of tumor cells is their ability to undergo metabolic reprogramming, which allows them to fulfill their high biosynthetic demands and sustain unchecked growth. In contrast, normal differentiated cells utilize mechanisms, such as oxidative phosphorylation, to generate the energy and biomass necessary for cellular processes. However, unlike normal tissues, most cancer cells alter their metabolism to primarily rely on aerobic glycolysis after the energy regulation and reprogramming process. This phenomenon is known as the Warburg effect. By providing ample sources of energy molecules, such as ATP and carbon intermediates for biosynthesis, the enhancement of aerobic glycolysis grants malignant cells a proliferative advantage [18].

However, tumor cells can bypass metabolic constraints by acquiring genetic mutations, such as tumor suppressors and oncogenes. These genetic changes can occur in cells throughout an individual’s lifespan and can alter the signaling pathways that control metabolic programming [19]. Abnormal changes in these signaling pathways lead to increased nutrient uptake and metabolism, which are necessary for producing the energy required for survival and cell proliferation. Energy metabolism, particularly glucose metabolism, has been linked to the control of growth by activating and silencing certain tumor genes, leading to uncontrolled cell proliferation, cell cycle arrest, and cell aging [20].

In this context, cancer cells exhibit alterations in glucose metabolism, displaying an increase in glucose uptake and glycolysis. This alteration in glucose metabolism and consumption results in a greater quantity of glycolytic metabolites and an increase in the amount of ATP generated by glycolysis. A majority of the carbon from the glucose, in the form of various glycolytic intermediates, enters multiple biosynthetic pathways. Most of the pyruvate generated during glycolysis is converted into lactate in the cytoplasm by the enzyme lactate dehydrogenase (LDH) and is secreted rather than oxidized in the mitochondria [21].

This metabolic change was first observed by Otto Warburg in 1924, who hypothesized that this metabolic alteration was specific to cancer cells and was caused by mitochondrial defects that resulted in the inhibition of the oxidative capacity of glucose to CO_2_. From this, he derived the hypothesis that dysfunctional mitochondria were re-sponsible for the development of cancer [22]. Although the Warburg effect is associated with cancer, different metabolic profiles have been associated with various types of cancer (see Table 1). The Warburg effect forms the basis for the use of positron emission tomography (PET) using 18-fluorodeoxyglucose (FDG), which accumulates pref-erentially in tumor cells due to their rapid uptake of glucose [23]. With regard to glucose metabolism, pyruvate from glucose glycolysis enters a truncated TCA cycle that ends in citrate, which is exported from the mitochondrial matrix to the cytosol [24]. Citrate is processed by the enzyme ATP citrate lyase (ACL) to produce acetyl-CoA, which can be used for fatty acid synthesis. This truncated TCA cycle results in a flux of metabolites out of the cycle (cataplerosis), which is necessary to balance the influx of metabolites (anaplerosis). In many types of cancer, glutamine is used in the TCA cycle [25].

Although glucose is the precursor of 90% of the lactate secreted by cancer cells, gluta-mine conversion provides approximately 40% of the intermediates in the TCA cycle and 30% of the generated ATP [26,27]. In normal tissue, approximately 10% of cellular energy is generated by glycolysis, while aerobic activity in the mitochondria contributes 90%. In tumor tissue, approximately 50% of cellular energy is generated by glycolysis, and the remaining energy is generated in the mitochondria. Thus, the alteration of the glycolytic metabolism of transformed cells is a crucial factor in cancer development and progression. Inhibiting glycolysis can reduce the proliferative advantage of cancer cells and induce cell death, making it a potential target for therapeutic intervention. However, the complexity of cancer metabolism and the potential for compensatory pathways make it challenging to selectively target glycolysis for therapeutic purposes. Further research is necessary to understand the specific metabolic changes in different types of cancer and to develop targeted therapies that can effectively inhibit glycolysis without causing negative side effects [28,29].

Authors agree that the reprogramming of cellular metabolism is influenced by the activation of oncogenes and inactivation of tumor suppressor genes. There is evidence to suggest that proto-oncogenes and tumor suppressor genes play a role in regulating metabolism [30]. For instance, the oncogene MYC can increase the Warburg effect, while the tumor suppressor gene p53 blocks this same effect, highlighting the regulatory activity of these molecules in the metabolism and their impact on cell proliferation [31]. This indicates that alterations in cellular metabolism, including changes in signal transduction pathways and metabolic adaptations, can be clonally selected during tumorigenesis and should be considered a central aspect in the development and growth of a tumor [27] (See Table 1).

**Table 1:** Metabolic effects of the main proteins in cancer cells. Adapted with permission from Valle-Mendiola and Soto-Cruz [27].

Protein	Expression in Cancer	Effects on the Metabolism
Pyruvate kinase isoform M2 (Isoform embryonic)	Increased expression in tumors and cancer cell lines	Increased glycolysis
Monocarboxylate transporters (MCTs)	Overexpressed in ovarian, prostate, gastric, and cervical cancers	Increased glycolysis
Glutaminase and glutamate oxaloacetate transaminases	Increased expression in cancer	Use of glutamine as a source of ATP and generation of TCA intermediaries
Via PI3K/Akt	Dysregulated in cancer	Increased glycolysis
HIF-1α	Overexpressed in cancer	Increased glycolysis
Myc	Dysregulated in cancer	Promotes the use of glutamine, glycolysis augmented
p53	Mutated in cancer (inactivated)	Active p53 inhibits glycolysis and promotes oxidative phosphorylation. Loss of p53, increased glycolysis
Phosphofruct kinase/fructose-2,6-biphosphatase gene B3 (six isoforms)	Augmented expression	Increased glycolytic flux
Hexokinase II	Increased expression in hepatoma, cervical cancer	Increased glycolysis
Phosphofruct kinase 1	Hyperactivated in cancer	Increased glycolysis
Pyruvate dehydrogenase kinase (PDK, four isoforms)	Increased in cancer	Increased glycolysis
Snail (E-cadherin repressor)	Increases epithelial–mesenchymal transition	Suppresses oxidative metabolism in mitochondria
Kisspeptin	Cancer metastasis suppressor of thyroid, ovary, bladder, gastric, esophageal, pancreatic, lung, pituitary, and melanoma	Promotes oxidative metabolism, inhibiting glycolysis

Authors agree that the reprogramming of cell metabolism is influenced by activated oncogenes and inactivated tumor suppressors. Evidence suggests that proto-oncogenes and tumor suppressor genes play a role in regulating metabolism [30]. For example, the oncogene MYC can enhance the Warburg effect, while the tumor suppressor gene p53 blocks this same effect, highlighting the regulatory activity of these molecules in metabolism and their influence on cell proliferation [31]. This suggests that alterations in cell metabolism, including changes in signal transduction pathways and metabolic adaptations, can be clonally selected during tumorigenesis and must be considered a central aspect in the development and growth of a tumor [27].

## 3. Mitochondrial Fitness and Cancer

In recent years, research into mitochondria and their roles in relation to exercise, diet, and disease has reached a peak. Generally, this research opens new pathways for understanding cancer’s biology by focusing on various elements of mitochondrial metabolism and function [16,32,33].

Mitochondria are widely recognized as the primary energy source in cellular metabolism, with ATP production and driving respiration being just one of their many functions. These functions also include the enhancement of an effective anti-viral host defense, regulation of their numbers and quality through mitochondrial biogenesis and mitophagy, regulation of their localization and metabolic activity through trafficking and dynamics, and performing cell signaling functions (including innate immune system signaling). As essential organelles, mitochondria must be continuously monitored to ensure their functional integrity. The combination of their integrity, efficiency, and dynamic response to stresses is commonly referred to as “mitochondrial fitness”. There is empirical evidence linking the loss of these functions to the development of cancer cells [34,35]. In addition, the gradual transformation of normal cells into increasingly malignant variants, known as tumorigenesis, can alter mitochondrial activities ranging from suppression of apoptosis to metabolic dysfunctions [36,37]. In sum, mitochondrial abnormalities and tumorigenesis can be caused by mitochondrial depolarization, oxidative stress, a lack of growth factors, or DNA damage.

Due to the critical role of apoptosis in maintaining healthy tissues by ensuring controlled cell death or proper balance between cell death and proliferation [38,39], reduced apoptosis appears to contribute to the onset and progression of tumors and malignancies [38]. In the case of melanoma metastases, the most prevalent malfunction is the loss of TP53 tumor suppressor activity, which encodes p53 and eliminates this essential damage sensor from the apoptosis-inducing circuitry [37,40], playing a crucial role in cancer evolution [41]. The P53 mutations in cancer patients across 20 tissues are the most frequent of any known gene (36.1%), further supporting the central role of TP53 in carcinogenesis [42]. Additionally, dysregulation of numerous proteins, such as the Bcl-2 family of proteins, which can exert pro- or anti-apoptotic activity in the cell, has been linked to the irregular death of tumor cells [7,38]. For example, elevated expression of Bcl-2 protein protects prostate cancer cells from apoptosis [43], and Mortenson et al. found that overexpression of Bcl-2 led to more activation of signaling molecules in pancreatic cancer cells [44].

In parallel with these functional insufficiencies in mitochondrial permeability transition [45], Porporato et al., 2018 highlighted that “malignant transformation” can continue to occur through at least two major mechanisms, as follows: the upsurge of potentially oncogenic DNA defects and the activation of potentially oncogenic signaling pathways through mitochondrial reactive oxygen species (ROS) [46,47], and the anomalous up-surge of fumarate, succinate, and 2-hydroxyglutarate (2-HG), distinctive mitochondrial metabolites [48]. One of the defining characteristics of this transformation of the tumor cell is the acquisition of an essentially glycolytic metabolism [47,48].

Metabolic reprogramming, a characteristic that plays a key role in the changes that define the phenotype of a tumor cell [37], enables cancer cells to meet their high biogenic demands for rapid, uncontrolled growth. While normal cells generate energy through oxidative phosphorylation, most cancer cells exhibit changes in nutrient metabolism and rely on aerobic glycolysis, also known as the Warburg effect [49,50]. This altered metabolism, once thought to be unique to cancer cells and caused by abnormal mitochondria, is now known to be essential for cancer cell activity as the deletion of cancer cell mitochondrial DNA (mtDNA) slows their growth rate and tumorigenicity [51]. The increased aerobic glycolysis in malignant cells provides a proliferative advantage by producing energy sources, such as ATP, and carbon intermediates for biosynthesis [52]. Tumor cells can bypass metabolic restrictions through the accumulation of mutations in key genes, such as tumor suppressors and oncogenes. These genetic mutations can accumulate in cells throughout a person’s life and affect the signaling pathways that control metabolic programming [53,54]. Furthermore, MtDNA mutations, found in various cancers, appear to alter mitochondrial metabolism, and promote tumorigenesis while allowing cancer cells to adapt to changing environments [55,56]. More recently, the functional relevance of mtDNA variation has been demonstrated in oncocytoma and prostate cancer [57].

In addition to mutations that directly affect mtDNA, mutations in nDNA-encoded mitochondrial enzymes have been identified in certain carcinomas [4,58]. Despite the extensive research on the role of mitochondria in cancer formation and progression, there is still much to be learned about this process. The quantity and quality of mitochondria, which are essential for metabolic function, are controlled and maintained through the mitochondrial life cycle and fitness, from the biogenesis of new mitochondria to the removal of dysfunctional mitochondria. As metabolism plays a significant role in cancer progression, it is reasonable to believe that inhibiting its metabolic pathway could inhibit tumor growth. However, as Oshima et al. found, this is not a straightforward task [59]. Exercise, on the other hand, appears to offer protection against the disease, reducing the risk of cancer by 10 to 25% [60].

## 4. Oxidative Stress

Numerous substances are produced during oxidative metabolism, which generates energy in the mitochondria through aerobic respiration. While most of these compounds are beneficial, less than 5% of them may be harmful to cells if their concentration increases [59,60,61]. These low-concentration products of oxidative metabolism are essential for subcellular processes, such as signal transduction, enzyme activation, gene expression, and disulfide bond formation during protein folding in the endoplasmic reticulum. Peroxisomes and enzymes, such as the detoxifying enzymes from the P450 complex, xanthine oxidase, and the nicotinamide adenine dinucleotide (NADPH) oxidase complexes that make up the NOX family, are sources of internal oxidative stress, much of which originates in the mitochondria [61]. Specifically, reactive oxygen species (ROS), such as the superoxide anion (O^2−^), hydrogen peroxide (H_2_O_2_), hydroxyl radical (OH^−^), singlet oxygen (^1^O_2_), and ozone (O_3_) [62,63], can cause oxidative stress when the balance between pro-oxidants and antioxidants is disrupted, leading to the alteration and damage of intracellular molecules, such as DNA, RNA, lipids, and proteins [64,65].

Recent research suggests that reactive oxygen species (ROS) can play a role in the development of cancer through a variety of mechanisms, including inducing inflammation, evading the immune response, regulating signaling pathways that control autophagy and apoptosis, promoting angiogenesis, and increasing drug resistance [66,67]. While there are currently few biomarkers that can clarify the specific role of oxidative stress in cancer pathogenesis [66], it has been suggested that a “chain reaction” can occur when oxidative damage to both mitochondrial and nuclear DNA leads to mutations that obstruct the oxidative phosphorylation pathway, resulting in increased ROS production, genomic instability, and the development of cancer [66,68,69].

### 4.1. ROS, Initiation, Promotion, and Progression of Cancer

Reactive oxygen species (ROS) have been linked to the initiation, promotion, and progression of cancer. During oxidative metabolism, which generates energy in the mitochondria through aerobic respiration, a small percentage of the substances produced may be harmful to cells if their concentration increases. These low-concentration products of oxidative metabolism, known as ROS, include the superoxide anion (O^2−^), hydrogen peroxide (H_2_O_2_), hydroxyl radical (OH^−^), singlet oxygen (^1^O_2_), and ozone (O_3_). When the balance between pro-oxidants and antioxidants is disrupted, oxidative stress occurs, leading to the alteration and damage of intracellular molecules, such as DNA, RNA, lipids, and proteins.

Furthermore, ROS have been shown to increase tumorigenesis through a variety of mechanisms, including inducing inflammation, evading the immune response, regulating signaling pathways that control autophagy and apoptosis, promoting angiogenesis, and increasing drug resistance. Additionally, it has been suggested that a “chain reaction” occurs when oxidative damage to both mitochondrial and nuclear DNA leads to mutations that obstruct the oxidative phosphorylation pathway, resulting in greater ROS production, genomic instability, and the development of cancer [66,67,68,69].

Inflammatory cells, such as those found in the lung cancer studies by Thannickal [70] and more recently by Aggarwal and collaborators [71], produce more ROS by inducing oxidant-generating enzymes, such as NADPH oxidase, iNOS, xanthine oxidase (XO), and myeloperoxidase (MPO). In chronically inflamed cells, the high secretion of ROS/reactive nitrogen species (RNS) attracts more activated immune cells, thereby amplifying dysregulated processes. Genetic and/or epigenetic alterations cause the dysregulation of oncogenes and tumor suppressor genes, while the large amount of cellular ROS/RNS produced is sufficient to overwhelm the endogenous antioxidant response. Therefore, oxidative stress and inflammation are closely linked, and failure to block these processes can lead to carcinogenesis [66].

Furthermore, damage to signaling pathways is particularly important during the tumor promotion and proliferation phases. The epidermal growth factor receptor signaling pathway is one example. Oxidative stress can affect crucial proteins, such as nuclear factor erythroid 2-related factor 2, RAS/RAF, the mitogen-activated protein kinases ERK1/2 and MEK, phosphatidylinositol 3-kinase, phospholipase C, and protein kinase C [72]. Additionally, cell survival is promoted by the oxidation and inactivation of PI3K/Akt signaling negative regulators, such as the phosphatases PTEN and PTP1B. The tumor suppressor PTEN is reversible inactivated by H2O2 in various cancers, such as glioblastomas, endometrial, breast, thyroid, and prostate cancers [73].

Protein dysfunctions may be responsible for the most DNA damage caused by hydroxyl radicals, which can generate mutations, genetic instability, and epigenetic changes that can lead to cancer. In fact, the DNA damage marker 8-OHdG is mutagenic, and high levels of 8-OHdG have been found in various cancers and are associated with a poor prognosis for ovarian cancer-specific survival [74]. Here, BRCA1, a tumor suppressor gene that is frequently mutated in many cancers, is a caretaker gene responsible for DNA repair and helps cells to cope with oxidative stress. It regulates the activity of the transcription factors Nrf2 and NFkB and can upregulate several antioxidant response genes. Here, NFkB plays a significant role in cell proliferation, cell survival, cell cycle regulation, and drug resistance. In this regard, tumor cells adapt to hypoxia and respiratory injury to resist chemotherapy by activating glucose metabolism, making them less sensitive to it [75].

The ROS also play a role in the regulation of cell survival and cell death pathways, such as autophagy or apoptosis [76]. Autophagy is an early line of defense against oxidative stress damage and increases when ROS levels rise. Dysregulation of autophagy has been linked to both tumor progression and therapy resistance. On the other hand, over-activation of autophagy can lead to cell death, a process known as autophagic cell death or type II programmed cell death. Similarly, ROS can also modulate the intrinsic apoptotic pathway, by triggering the release of pro-apoptotic molecules from the mitochondria and activating caspases, leading to cell death.

In summary, ROS play a crucial role in the development of cancer through a variety of mechanisms, including inducing inflammation, evading the immune response, regulating signaling pathways that control autophagy and apoptosis, promoting angiogenesis, and increasing drug resistance. Additionally, it has been suggested that a “chain reaction” occurs when oxidative damage to both mitochondrial and nuclear DNA leads to mutations that obstruct the oxidative phosphorylation pathway, resulting in greater ROS production, genomic instability, and the development of cancer. Therefore, targeting oxidative stress and inflammation may be a potential strategy for cancer therapy.

### 4.2. Tumor Death by ROS Control

Endogenous and exogenous antioxidants can prevent and repair damage caused by reactive oxygen species (ROS). These substances, known as “free radical scavengers”, can improve the immune defense and lower the risk of disease and cancer [77,78,79]. Increasing ROS levels to toxic levels and exhausting the antioxidant system capacity can cause programmed cell death, making the antitumorigenic signaling of ROS a potential target for cancer therapy [80].

Chemotherapy agents, such as doxorubicin, which is used to treat a variety of cancers including breast, esophageal, and endometrial carcinomas, work by increasing ROS production, activating the tumor suppressor p53, and ultimately leading to tumor cell death [81]. A treatment course of arabinocytosine, which hinders DNA replication, followed by anthracyclines to increase ROS, has also been shown to have beneficial effects for patients with acute myeloid leukemia [80,82].

Plant-derived foods, such as those rich in polyphenols, have been shown to have beneficial effects on cancer through various mechanisms including the regulation of nuclear factor kappa B (NFkB) expression and chromatin remodeling, and the stimulation of apoptotic cell death in preneoplastic or neoplastic cells through the activation of cyto-chrome c and caspases, the arrest of the cell cycle, and the modulation of signaling pathways (NFkB, JAK/STAT) [83,84].

To develop selective and effective cancer treatment strategies, a better understanding of how ROS regulates autophagy and apoptosis is necessary. By analyzing the role of increased ROS production in cancer, ROS-regulated signaling pathways, and specific antioxidant targets, it is possible to develop targeted therapies that either induce or inhibit ROS depending on the molecular context and microenvironment of the cancer.

## 5. Apoptosis

Apoptosis, a process of programmed cell death, has been studied extensively in relation to metabolic health due to its role in regulating various physiological processes [39]. Alterations in the apoptosis process have been linked to neurodegenerative diseases, such as Alzheimer’s, ischemic damage, autoimmune disorders, and cancer [39,85,86,87]. Mitochondrial health and apoptosis are closely connected, as mitochondrial activity, which involves one of the most important metabolic processes, oxidative phosphorylation (OXPHOS), is also involved in cell respiration [88]. Mitochondria also modulate redox status and the production of reactive oxygen species (ROS) which contribute to apoptosis induction [89]. The ROS can also intensify oxidative stress, leading to cellular damage and negative effects on proliferation, metabolism, genetic transcription, and apoptosis control [90]. The mitochondria play an important role in the regulation of the intrinsic apoptotic pathway, as previous authors have pointed out [91].

Once activated, cell death signaling pathways lead to modification of the outer mitochondrial membrane, increasing its permeability and allowing the passage of various substances, such as cytochrome c, endonucleases, and other intermembrane space proteins from mitochondria to the cytosol. These proteins activate caspase signaling, which plays an important role in promoting the apoptosis pathway [92,93]. Caspases are involved in various processes, such as blebbing of the plasma membrane, loss of cell volume, synthesis of apoptotic bodies, and degradation of chromosomal DNA [94]. This ultimately leads to the destruction of cell corpses and the end of cellular degradation [95]. The association between cancer progression and mitochondrial dysfunction was first proposed by Otto Warburg nearly a century ago, when he observed active glucose metabolism in cancer cells, called aerobic glycolysis, suggesting a possible link between the two [1,96,97].

Recent research has also proposed that cancer development may be affected by other mitochondrial disorders, such as mitochondrial DNA mutations, mitochondrial enzyme defects, ROS generation, and changes in oncogenes or tumor suppressor levels [98]. It is now understood that alterations in various biochemical functions including the tricarboxylic acid cycle, mitochondrial DNA mutations, and respiratory chain dysfunction, in addition to subsequent mitochondrial oxidative stress, may cause resistance to apoptosis and contribute to cancer progression [99]. The relationship between mitochondrial dysfunction and cancer development may also be bidirectional, as the current literature suggests that oncogenes may be responsible for initiating mitochondrial DNA mutations, mitochondrial alterations, ROS level increase, and induction of the apoptosis process [100,101,102,103,104].

## 6. Inflammation Response

The importance of the inflammatory response in mitochondrial functioning and its influence on cancer development has been well documented in the literature. Studies have shown that inflammatory diseases may be related to an excess of reactive oxygen species (ROS), leading to mitochondrial dysfunction. This mitochondrial alteration may also increase the production of ROS, creating a vicious cycle of the inflammatory process [105,106]. Inflammatory mediators, such as tumor necrosis factor alpha (TNFα) and interleukin 1 beta (IL-1β) and nitric oxide (NO) have been identified as potential contributors to mitochondrial dysfunction [106,107].

Additionally, TNFα and IL-1β have been proposed to increase ROS production, reduce the activity of the mitochondrial respiratory chain and ATP production, and decrease the mitochondrial membrane potential [108,109]. Furthermore, TNFα has also been directly linked to mitochondrial injury by causing changes in mitofusin proteins, which have been associated with structural changes in mitochondria [110]. Meanwhile, NO has been associated with oxidative events, as it can form peroxynitrite, a harmful oxidant, through its union with super-oxide anion. This can result in oxidative damage to mitochondria, possibly through the inhibition of various types of mitochondrial enzymes [107,111]. Furthermore, NO also decreases the mitochondrial membrane potential and blocks mitochondrial respiration by reducing the activity of complexes III and IV of the electron transport system. Alterations in both complexes have been linked to various types of cancer [108,112].

Increased production of ROS has been found to be linked to numerous cancerous events due to increased intracellular oxidative stress [113]. Therefore, ROS can be associated with the activation of oncogenes and the inhibition of tumor suppressors [114]. Additionally, ROS have also been linked to a reduction in p53, as well as an increase in the expression of nuclear factor kappa B (NF-kB)-dependent inflammatory genes, including cytokines. Research has shown that the induction of cytokines and the subsequent expression of NF-kB can enhance tumor proliferation [115], cancer progression [116], the establishment of the pre-metastatic field [117], oncogenic recurrence, and increased resistance to therapy [118].

Furthermore, ROS have also been linked to other types of tumor suppressor proteins, such as p21, p16, and sirtuin-3 (SIRT3) [119]. Proteins p21 and p16 are responsible for blocking cyclin-dependent kinases (CDKs), which are involved in halting the cellular cycle and inhibiting DNA replication [120]. Therefore, if these proteins are reduced, cell proliferation is favored, promoting the development of tumor cells [120]. Here, SIRT3 is a mitochondrial deacetylase that plays a role in various mitochondrial functions, such as OXPHOS, the tricarboxylic acid cycle, and the urea cycle [121]. A decrease in these metabolic activities may compromise mitochondrial function and promote tumor development.

Furthermore, ROS synthesis in mitochondria has also been linked to the activation of the NLRP3 inflammasome, a cytosolic multiprotein complex responsible for activating the pro-inflammatory caspase and leading to the release of pro-inflammatory cytokines [122,123]. Therefore, inflammasome-mediated responses, such as IL-1β, may modulate cancer progression due to their ability to promote oncogenesis and inhibit tumor sup-pression [123]. Pro-inflammatory cytokines have been linked to the regulation of cancer cells and appear to be involved in various processes, such as proliferation, invasiveness, neoangiogenesis, neurogenesis, and fibrogenesis [124].

Some studies have suggested that certain tumors show an increase in the number of inflammatory precursors, leading to pro-oncogenic inflammation and improving the tumor microenvironment, creating conditions that promote tumor development. In this case, the inflammatory response may not only contribute to the development of cancer but also to its progression. Additionally, pro-inflammatory cytokines may also play a role in cancer drug resistance, making it more difficult to treat the disease.

Therefore, understanding the relationship between inflammation, mitochondrial dysfunction, and cancer is crucial in developing new therapies to target these processes and improve the outcome for cancer patients [125,126,127,128,129,130].

## 7. Physical Activity, Cancer, and Stress

Cancer is the leading cause of death in high-income countries and the second leading cause in low- and middle-income countries [130]. Even though survival rates have improved in recent years, the adverse (short-term and long-term) effects of cancer and its treatment remain a concern [131]. The positive impact of physical exercise on cancer has been recognized by comprehensive reviews from the International Agency for Research on Cancer [132] and the World Cancer Research Fund [133] for various types of cancer. In this section, we will summarize key findings on the role of physical activity in cancer and stress.

Physical exercise has been shown to reduce the risk of certain cancers, such as colorectal, breast (post-menopause), endometrial, gastroesophageal, bladder, and prostate cancer, and to decrease the risk of cancer relapse and improve survival rates [134,135,136,137,138,139,140]. However, the evidence for its impact on blood cancer, pancreatic cancer, ovarian cancer, thyroid cancer, and liver cancer is limited [141,142]. The beneficial effects of physical activity on cancer are largely mediated through its effects on the immune system and inflammation [60,143].

Regular moderate physical exercise enhances immune function, while a lack of physical activity (sedentarism) and very intense exercise may suppress immune function and increase the risk of cancer [142]. However, most of these studies have been conducted in vitro using cultured malignant cells, rather than the patient’s own tumor cells [143].

Physical activity, both acute and regular, affects innate immunity, which is our first line of defense against infectious pathogens and is involved in tissue repair. It is non-specific and does not have a memory. For example, natural killer (NK) cell surveillance increases during recovery from aerobic or anaerobic exercise among cancer patients and healthy individuals [144,145]. Exercise can increase the number of NK cells in the circulating blood by up to five times compared to before exercise [145]. However, extremely intense and exhausting exercise may impair NK cell activity or function, as the number of circulating NK cells decreases when they enter sites of muscle damage [145,146].

On the other hand, acute, intense physical activity may impair acquired immunity (also known as adaptive or specific immunity), which is a specialized form of immunity with memory that improves with repeated exposure [142]. Acquired immunity is specialized in fighting infections and involves antigen-presenting T helper (CD4+) lymphocytes and the major histocompatibility complex class II. However, this impairment is only temporary unless recovery is insufficient (usually within 24 h) due to prolonged, intensive physical exercise and insufficient time to recover [147]. In summary, physical exercise regulates the immune environment by stimulating NK cells and the proliferation of T cells [148]. However, only moderate physical exercise inhibits the growth of cancer cells by inducing apoptosis [142].

In addition, regular moderate physical exercise has anti-inflammatory effects, which are largely responsible for its benefits [149]. Human cross-sectional studies support that regular moderate physical exercise releases anti-inflammatory cytokines and reduces the production of pro-inflammatory cytokines or cancer biomarkers, such as tumor necrosis factor-alpha (TNFα), interleukin-6 (IL-6), and C-reactive protein (CRP) [131,150]. Chronic inflammation is a well-established risk factor for cancer [151].

Physical inactivity leads to the accumulation of visceral fat and the activation of pro-inflammatory biomarkers, leading to chronic inflammation, which is not only involved in tumor growth but also other serious health issues, such as insulin resistance, atherosclerosis, and neurodegeneration, also known as the “disease of physical inactivity” [152].

Low-calorie diets, as with physical exercise, have anti-inflammatory effects, which makes sense, since diet and physical activity are both related to energy balance, or the ratio of caloric expenditure to caloric intake [153,154]. Energy balance is directly linked to the risk of cancer, recurrence, and survival [140,153,155,156]. Being overweight or physically inactive may account for up to 26% of the total risk of colorectal cancer [157]. Additionally, those with a body mass index (BMI) higher than 40 have twice the risk of death from cancer compared to those with a BMI lower than 25 [153].

Third, physical activity also reduces cancer recurrence and improves prognosis, including health outcomes and survival rates [140,144,158,159,160]. Physical exercise improves the cancer microenvironment by increasing blood flow and activating immune cells, limiting cancer cell growth [142,148,154]. Additionally, physical exercise also reduces the adverse effects of cancer and cancer treatment, such as fatigue, by reducing oxidative stress and improving cardiovascular and cognitive function [142,161]. However, further research is needed to fully understand the relationship between metabolism and immune regulation during exercise.

It is worth noting that the impact of physical exercise on the immune system and inflammation is mediated by the hypothalamic pituitary adrenal pathway (HPA) and sympathetic nervous system, which are involved in the stress response [151]. Therefore, chronic physical inactivity acts as a chronic stressor that may increase the risk or progression of several cancers, while regular moderate physical activity has the opposite beneficial effect on cancer and can serve as a stress-reduction intervention [153,162]. Psychological stress or the perception of a lack of control (helplessness) can release stress hormones that increase the risk of tumor growth and metastasis, while beta blockers may decrease rates of recurrence (for example, in women with breast cancer) [162,163,164].

Furthermore, both psychological stress and physical inactivity lead to the accumulation of visceral fat and the activation of a network of inflammatory pathways that result in chronic inflammation, which is involved in tumor growth, insulin resistance, atherosclerosis, neurodegeneration, and other stress-related diseases that are associated with physical inactivity [151,162,165,166,167].

Future research should further examine the impact of the four dimensions that form the foundation of physical activity or exercise prescription, known as the FITT principle (frequency, intensity, duration, and type) [168], on cancer. Additionally, since most studies are based on cohort and case-control designs, it is recommended to use longitudinal studies and randomized controlled trials (RCTs).

In conclusion, physical exercise should be considered an important intervention for preventing and treating cancer and its complications, as it can reduce cancer incidence by inhibiting cancer growth and metastasis and improving the adverse effects of cancer and cancer treatment. The protective effect of regular physical exercise against cancer and other stress-related diseases is due to its ability to reduce visceral fat mass and its anti-inflammatory effects. Indeed, long-term chronic stress also weakens the immune system.

These findings have important implications for public health and could lead to the development of tailored and novel exercise prescriptions for cancer patients, focusing on primary prevention rather than curing cancer in advanced stages [153,169,170,171]. The American Cancer Society already endorses the physical activity recommendations of the College of Sports Medicine, which emphasize the importance of building up at least 150 min of moderate-intensity aerobic physical exercise or 75 min of vigorous intensity per week [130]. This is particularly important now, as the World Health Organization estimates that fewer than 25% of adults follow the recommended 150 min of moderate physical exercise per week, especially in high-income countries [172].

## 8. Effect of Aerobic and Resistance Exercise Interventions on the Cancer Treatment Continuum

There is strong evidence showing that physical activity is associated with a reduced risk of several cancers, including breast, colon, bladder, endometrium, stomach, kidney, esophagus, and lung [130], and that it can inhibit tumor growth [173].

Exercise has been shown to reduce tumor incidence, growth, and metastasis in rodent tumor models [174]. International medical oncology societies recognize exercise pro-grams as a co-adjuvant therapy and an essential part of treatment for cancer patients, based on scientific evidence [175,176,177,178,179,180]. Exercise can also positively reduce symptoms of chronic diseases and alleviate the adverse effects of cancer treatments, such as cancer fatigue, pain, sleep disorders, anxiety, and depression, and overall improve quality of life [181]. There have been studies that have analyzed the effect of pre-surgical exercise on physiological, functional, and health markers and variables related to post-surgical recovery [175,182].

Available evidence suggests that pre-surgical aerobic and resistance training, either in combination or alone, can benefit cancer patients by improving cardiovascular fitness, muscle strength, and quality of life [175,182]. Pre-surgical exercise may also reduce the length of hospital stay [182]. Exercise interventions have included high-intensity training (cycling 5 days per week for 30 min at 50–100% of VO2max) [183] or moderate aerobic exercise (3 h per week) in combination with strength training (40 min per week) [184]. Based on existing exercise guidelines for cancer patients [185,186,187,188,189,190], pre-surgical exercise should include endurance exercise based on high-intensity interval training (60–90% of VO_2max_) in a 20–35-min session and strength exercise using global exercises (using the patient’s body weight or 0–40% of 1RM) performing 2 sets of 10 repetitions and including passive or active stretches for all body parts (30 s per exercise).

Improved sleep quality is a factor that can be affected by fatigue, which is commonly experienced by cancer patients. Sleep disturbance and clinical insomnia are common among cancer patients [191,192,193,194,195,196], and exercise has been shown to have a positive impact on sleep quality in these individuals. This is likely due to the improvement in thermoregulation and energy conservation that results from exercise, as well as a reduction in proinflammatory cytokines and the increase in plasma concentrations of sleep mediators [197,198,199,200,201,202].

Studies have also shown that aerobic exercise has a positive effect on improving sleep outcomes in cancer patients, and that this effect can be sustained for up to 6 months after an exercise intervention program [199]. Resistance exercise has also been shown to effectively improve sleep quality in cancer patients [203]. However, the positive effect of isolated resistance exercise on sleep quality seems to be attenuated when combined with aerobic exercise, as compared to aerobic exercise alone [203]. It is worth noting that the effect of exercise interventions on sleep quality is enhanced with higher frequency, intensity, and duration of the program [203].

In summary, exercise interventions, including aerobic and resistance training, have been shown to be effective in improving cardiorespiratory fitness, muscle strength, fatigue, and sleep quality in cancer patients. However, it is important to note that more research is needed to determine the specific effects of exercise on various types of cancer, as well as to develop evidence-based guidelines for exercise interventions in cancer patients.

Improved sleep quality is a crucial factor that can be affected by fatigue, which is commonly experienced by cancer patients. Research has shown that cancer patients often experience sleep disturbance and clinical insomnia [199]. However, exercise has been shown to have a positive impact on sleep quality in these individuals.

The positive impact of exercise on sleep quality is likely due to the improvement in thermoregulation and energy conservation that results from exercise, as well as the re-duction of proinflammatory cytokines and the increase in plasma concentrations of sleep mediators [200,201,202]. Studies have found that aerobic exercise has a positive effect on improving sleep outcomes in cancer patients, and that this effect can be sustained for up to 6 months after an exercise intervention program [199]. Additionally, resistance exercise has also been shown to effectively improve sleep quality in cancer patients [203].

It is worth noting that when resistance exercise is combined with aerobic exercise, the benefits of isolated resistance exercise on sleep quality may be attenuated as compared to aerobic exercise alone [203]. Furthermore, higher frequency, intensity, and duration of an exercise program have been associated with greater improvements in sleep quality [203].

Fatigue, a common symptom among cancer patients, can be significantly impacted by sleep quality. Cancer patients often experience sleep disturbance and clinical insomnia [199], and evidence suggests that exercise can have a positive effect on sleep quality. This may be due to improved thermoregulation and energy conservation, as well as reduced proinflammatory cytokines and increased plasma concentrations of sleep mediators [200,201,202]. Aerobic exercise, in particular, has been shown to improve sleep outcomes in cancer patients, with effects lasting up to 6 months after an exercise intervention [199]. Resistance exercise has also been shown to effectively improve sleep quality in cancer patients [203], but the benefits may be attenuated when combined with aerobic exercise, compared to aerobic exercise alone [203]. Additionally, higher frequency, intensity, and duration of an exercise program have been associated with greater improvements in sleep quality [203].

The goal of cancer treatment includes not only reducing the size of the tumor and im-proving survival rates, but also improving the quality of life [204], which is a predictor of survival [205]. Exercise interventions have been shown to be effective in improving the overall quality of life in cancer patients [206]. Both aerobic and resistance training have been shown to improve physical, role, and global quality of life [207]. A previous meta-analysis found that emotional quality of life is improved with aerobic exercise, but there were no changes observed in social and cognitive quality of life [207]. Additionally, high-intensity training was found to be less effective at improving quality of life compared to moderate-intensity training [207].

Depression is another important factor that can affect the quality of life in cancer patients, and it is a common side effect of cancer and its treatment [208]. Depression is linked to a lower quality of life, can reduce adherence to treatment, and increases the risk of relapse and mortality in cancer patients [208]. The prevalence of depression varies depending on the type of cancer, ranging from 7–9.3% in Hodgkin lymphoma to 25–52% in head and neck cancer [209,210]. Exercise interventions, along with other non-pharmacological treatments, have been shown to reduce symptoms of depression in cancer patients [211] and have effects similar to those of drugs or psychotherapy, specifically in mild to moderate depression [212]. A previous meta-analysis [208] reports that exercise interventions produce small improvements in depressive symptoms in cancer survivors. An umbrella review that included 20 meta-analyses [188] examining depression in cancer patients concluded that exercise (including both aerobic and resistance exercise) has a beneficial effect on depression in cancer survivors. Exercise sessions lasting more than 30 min and performed five times per week have been found to have a larger effect on reducing symptoms of depression [212].

The symptom of cancer cachexia, characterized by ongoing loss of skeletal muscle mass with or without loss of fat mass and a progressive decrease in physical functioning [213], can be managed with exercise that modulates muscle metabolism, insulin sensitivity, and inflammation [214,215]. However, a previous Cochrane review [216] that included four randomized controlled trials suggests that the effectiveness, acceptability, and safety of exercise for adults with cancer cachexia is unclear. Specifically, there is low-certainty evidence of the effects of exercise (strength training) or exercise plus other interventions (multimodal intervention) on lean body mass. In addition, the evidence of the effects of exercise on muscle strength, fatigue, submaximal exercise capacity, or health-related quality of life in adults with cancer cachexia is also low-certainty. The conclusions about the effectiveness of exercise on cancer cachexia should be updated with the inclusion of high-quality randomized controlled trials.

Despite this, the benefits of exercise for cancer patients are well-established. However, it is important to ensure that exercise interventions are safe and feasible, particularly for those with advanced cancer. A systematic review [217] suggests that exercise interventions are generally safe, with a low incidence of adverse effects. The most common adverse event related to exercise is injury or musculoskeletal pain, which has been reported to occur at a rate ranging from 0–25.8% in a previous review [188]. This high-lights the importance of professional programming and supervision during exercise interventions to reduce the risk of such adverse effects.

Exercise is a crucial aspect of cancer treatment, and guidelines recommend a concur-rent exercise program that includes endurance, strength, and stretching training [185]. The intensity and frequency of the exercise should be tailored to the patient’s previous training level and current health status, but typically involves 3 days per week of moderate to high-intensity endurance training (ranging from 41–64% to 80–90% of VO2peak) for 30–40 min, as well as 2 days per week of strength training consisting of 2 sets of 10 repetitions using light weights (40–60% of 1RM) and all-body stretching exercises (30 s per exercise) [186]. It is recommended that patients rest for 2 days after high-intensity training [186].

These recommendations also apply to cancer survivors [185,186]. However, barriers, such as a lack of specialized rehabilitation services, low awareness, and a lack of capacity to offer physical exercise rehabilitation programs, can hinder the implementation of exercise interventions for cancer patients. In addition, patient-specific inconveniences, such as the time of day or location of the exercise program, may also pose a problem. [218] Home-based exercise interventions have been shown to be an effective alternative for cancer patients who face accessibility barriers to traditional center-based exercise programs. [218] These interventions are feasible and provide health benefits for cancer patients during rehabilitation [218].

In summary, exercise intervention is crucial in managing physiological and physical factors, improving quality of life, reducing fatigue, and addressing mental health concerns throughout the cancer treatment journey. It can also help to alleviate negative side effects.

## 9. Active Life Behaviors

According to recent studies, approximately 30–40% of cancers are potentially preventable [219,220,221,222]. Physical inactivity, sedentary behavior, and obesity are among the major risk factors for cancer that can be modified by individuals [219,220]. The prevalence of these risk factors, as defined by low levels of physical activity, sedentary behavior, and obesity, is currently high [221,223]. The National Cancer Institute of the United States cites extensive research showing that increased physical exercise is associated with a reduced risk of cancer. Although observational studies cannot establish a causal relationship, there is a plausible mechanism for such a relationship and consistent findings across diverse populations. Research and meta-analyses have linked higher levels of physical activity to several types of cancer, including bladder, breast, colon, uterine, and esophageal cancer [224].

It is well established that being overweight, which is often associated with sedentary lifestyles and physical inactivity, is linked to chronic diseases. This is supported by studies from Turkey, which found that only half of the patients diagnosed with colon or breast cancer between 2017 and 2018 were physically active, while the rest had higher BMIs and were more likely to be physically inactive and at a more advanced stage of the disease. Diet, gender, and health habits, such as smoking, are also associated with cancer [225]. To more clearly address the relationship between these factors and the various types of cancer, we will divide the discussion into the following sections.

### 9.1. Colon Cancer

Colorectal cancer is the third most common type of cancer worldwide, affecting both men and women. Approximately 2 million new cases of colorectal cancer were reported in 2020 [226,227,228]. The 5-year relative survival rate for this type of cancer is 65%, but this varies based on the stage and location of cancer [229]. A meta-analysis conducted by Wolin et al. in 2009 included 52 studies and found an inverse association between physical activity and colon cancer, with a relative risk (RR) of 0.76 (95% confidence interval (CI): 0.72, 0.81). This association was found to be similar in both men (RR = 0.76, 95% CI: 0.71, 0.82) and women (RR = 0.79, 95% CI: 0.71, 0.88). The findings from case-control studies were more conclusive than those from cohort studies, supporting previous research that has shown an inverse association between physical activity and colon cancer in both men and women [230]. A meta-analysis by Liu et al. in 2016 found that outdoor physical activity had a protective effect against colorectal cancer, with an RR of 0.84 (95% CI 0.77 to 0.93). Similarly, long-term physical activity (LTPA) was found to have a protective role against colorectal cancer starting at a minimum of 10 MET-hours per week, with an RR of 0.92 (95% CI 0.85 to 1.00) [172].

### 9.2. Endometrial Cancer

Endometrial cancer is the 6th most common cancer in women and the 15th most common cancer overall. In 2020, there were over 400,000 new cases of endometrial cancer and approximately 100,000 deaths [231]. Strong evidence shows that higher levels of physical activity can reduce the risk of several types of cancer, including endometrial cancer, while moderate evidence suggests an inverse association between physical activity and endometrial cancer [231]. The most significant risk factors for developing this type of cancer are postmenopausal unopposed estrogen therapy, obesity, and nulliparity [232,233,234,235].

### 9.3. Esophageal and Stomach Cancers

In 2020, an estimated 605,100 people were diagnosed with esophageal cancer worldwide, and the 5-year survival rate for this type of cancer is 20% [236,237]. Physical exercise, particularly supervised training, has the potential to improve cardiorespiratory fitness, although most studies on this topic have focused on more common cancers, such as colon and endometrial cancer. A population-based study that examined the relationship between body mass index, physical activity, and sedentary behavior and the risk of esophageal adenocarcinoma (EA), esophageal squamous cell carcinoma (ESCC), gastric cardia adenocarcinoma (GCA), and gastric noncardiac adenocarcinoma (NGCA) found evidence of an inverse association between physical activity and the risk of NGCA.

However, there was no apparent relationship between body mass index and esophageal adenocarcinoma and physical activity or sedentary behavior [238]. Other research, including meta-analyses, has shown that the risk of esophageal adenocarcinoma is significantly reduced in physically active individuals compared to those who are physically inactive (RR = 0.79, 95% CI 0.66–0.94) [239]. In contrast, physical activity was not found to be associated with a lower risk of esophageal squamous cell carcinoma [134]. Additionally, a study that measured the association between high levels of physical activity and anatomic site and tumor histology found a risk reductions for esophageal adenocarcinoma (RR = 0.79, 95% CI = 0.66–0.94), gastric adenocarcinoma of the cardia (RR = 0.83, 95% CI = 0.69–0.99), and non-cardia gastric adenocarcinoma (RR = 0.72, 95% CI = 0.62–0.84). The relative risk of total gastroesophageal cancer for high versus low physical activity was 0.82 (95% CI = 0.74–0.90). This study concluded that there is an inverse relationship between physical activity, particularly exercise frequency, and gastroesophageal cancer risk [134].

### 9.4. Renal Cancer

In 2013, it was reported that people who engaged in intense physical activity had a significantly lower risk of developing kidney cancer than those who were less active [134]. People who reported engaging in “any physical activity” were 50% less likely to die from kidney cancer than those who did not exercise, while obese individuals with a BMI of 30 were nearly three times more likely to die than normal-weight individuals. Given the likely association between increased adiposity and cancer recurrence, survivors of kidney cancer (renal cell carcinoma, or RCC) need to maintain a healthy weight [240].

### 9.5. Lung Cancer

Currently, the epidemiological evidence about the relationship between physical activity and lung cancer risk is inconclusive. However, a meta-analysis of cohort studies found an inverse association between physical activity and lung cancer risk. Additionally, smokers who engaged in high levels of physical activity were associated with a 10% lower risk of lung cancer, although this association was not significant among nonsmokers [241]. These results are inconclusive and further intervention studies are needed to confirm these findings.

### 9.6. Other Cancers

Unlike other types of cancer, very few meta-analyses and systematic reviews have been published on the relationship between physical activity and hematological malignancies, and the findings are inconclusive. A study by Walter et al. found an association between regular physical activity and a dose-dependent risk reduction for most hematological malignancies, particularly myeloid malignancies, in US patients [242]. However, a study by Van Veldhoven et al. found no evidence of any effect of total physical activity on non-Hodgkin lymphoma (NHL) or NHL-B risk for men or women in European patients [243]. Another study by Teras et al. found no evidence of a statistical interaction between physical activity and sitting time, or between body mass index and physical activity or sitting time [239]. Despite these inconclusive findings, it is known that cancer is a significant public health problem and a high economic burden for countries. Lifestyle factors, such as physical activity, play a critical role in the prevention and, potentially, the prognosis of cancer.

There is strong evidence that physical activity before, during, and after cancer diagnosis can improve outcomes for certain types of cancer, particularly breast and colorectal cancer. Future studies are needed to strengthen the evidence and provide more detailed information on less common types of cancer. In the meantime, evidence-based recommendations to increase physical activity and decrease sedentary behavior in cancer survivors should be followed to improve the health of these individuals.

Finally, further studies are needed to measure the association between physical activity and all types of cancer, especially the less prevalent types.

### 9.7. Systemic Factors and Cancer

Several systemic processes act to regulate mitochondrial function, including the products of the gut microbiome and the circadian rhythm. Recent work indicates that a short-chain fatty acid, butyrate, optimizes mitochondrial function via the upregulation of sirtuin-3, and consequent deacetylation and disinhibition of the pyruvate dehydrogenase complex leads to an increase in the conversion of pyruvate to acetyl-CoA, resulting in increased ATP production from the TCA cycle and oxidative phosphorylation [244]. An increase in available acetyl-CoA can also be utilized as a necessary co-substrate for the enzymatic conversion of serotonin to N-acetylserotonin in the initiation of the mitochondrial melatonergic pathway, with melatonin providing antioxidant and wider protective effects on mitochondrial function [245]. Moderate exercise has positive impacts on the gut microbiome [246,247]. Pineal gland melatonin is a significant regulator of mitochondrial function at night, and its suppression over the course of aging and in shift workers will contribute to an increased risk of carcinogenesis [248]. Exercise can have positive effects on pineal melatonin production [249]. It is important to note that the benefits of butyrate and melatonin are not only on the mitochondria of cells that may be transformed into tumors but also on the cells that monitor and dispose of precancerous cells and emerging tumors, namely natural killer cells and CD8+ T cells [250]. Such exercise-induced changes in the gut microbiome and pineal melatonin will act on mitochondrial function across all body cells to better optimize their normal function and homeostatic interactions.

## 10. Conclusions

There is a growing body of evidence that suggests that proper mitochondrial function plays a critical role in cancer development. Healthy mitochondria are vital for maintaining metabolic processes and controlling cell apoptosis. When chronic inflammation occurs, the mitochondria may struggle to support and regulate the cells, leading to impaired tissue regeneration and damage to the affected organ. Both endurance and resistance exercise, as well as an active lifestyle, have been shown to improve mitochondrial function and may potentially reduce the risk or impact of cancer disease.
